# Network analysis of interpersonal conflict, emotional exhaustion and psychological distress among mental health nurses in the workplace: a cross-sectional survey

**DOI:** 10.3389/fpubh.2025.1559351

**Published:** 2025-05-30

**Authors:** Fangxinrui Qiu, Yuan Li, Chunfen Zhou, Yidan Sun, Jie Li, Jiao Tang

**Affiliations:** ^1^International Medical College of Chongqing Medical University, Chongqing, China; ^2^Department of Nursing, West China Second University Hospital/West China School of Nursing, Sichuan University, Chengdu, China; ^3^Key Laboratory of Birth Defects and Related Diseases of Women and Children (Sichuan University), Ministry of Education, Chengdu, China; ^4^Mental Health Center, West China Hospital, Sichuan University, Chengdu, China; ^5^Department of Toxicology/Nephrologyental, West China Fourth Hospital of Sichuan University, Chengdu, China; ^6^School of Nursing, Chongqing Medical University, Chongqing, China

**Keywords:** mental health nurses, interpersonal conflict, burnout, emotional exhaustion, psychological distress

## Abstract

**Background:**

Mental health nurses (MHNs) frequently engage in intense interpersonal interactions and encounter various forms of conflict with patients, colleagues, and their families. These conflicts can disrupt workplace harmony and significantly affect nurses' mental wellbeing. This study aims to analyze how workplace interpersonal conflicts affect nurses' emotional exhaustion and psychological distress through structural network analysis.

**Methods:**

A cross-sectional descriptive correlational survey was conducted using the Customer Interpersonal Injustice Scale, Interpersonal Conflict at Work Scale, Bi-directional Work-Family Conflict Scale, Maslach Burnout Inventory (emotional exhaustion sub-scale), and the 6-item Kessler Psychological Distress Scale. The survey was completed by 858 MHNs and 643 non-MHNs across six comprehensive hospitals and nine psychiatric hospitals from western China.

**Results:**

5.1% of all participants reported severe distress. Emotional exhaustion and psychological distress were associated with lower subjective social status, more severe conflicts with patients and supervisors, and bidirectional work-family conflict. Key risk factors for severe psychological distress included being an MHN, patient mistreatment, and bidirectional work-family conflict, while higher subjective social status was a protective factor. Network analysis showed no significant differences in conflict structures between MHNs and non-MHNs. Within the network, the work-to-family conflict most significantly impacted emotional exhaustion, while emotional exhaustion had the greatest influence on psychological distress.

**Conclusion:**

This study revealed that MHNs experienced a higher rate of severe psychological distress than non-MHNs, with emotional exhaustion and psychological distress closely associated with subjective social status and interpersonal conflict. Given that work-to-family conflict and emotional exhaustion were key nodes in the network, targeted interventions are urgently needed to alleviate psychological distress.

## 1 Introduction

Mental health nurses (MHNs) occupy a distinct position within the healthcare workforce, characterized by a heightened degree of emotional labor and interpersonal engagement. Their clinical responsibilities extend beyond conventional nursing duties, necessitating continuous therapeutic interactions with individuals experiencing severe mental health challenges. These interactions often involve managing unpredictable behaviors and emotionally charged situations, setting MHNs apart from their peers in other nursing specialties (1). Moreover, the emotional labor experienced in the workplace often extends its impact into the family (2). Consequently, MHNs are often exposed to various forms of interpersonal conflict, which can arise from interactions with colleagues, patients, and their families. The complexity and intensity of these conflicts have profound implications for MHNs' mental health, increasing their vulnerability to emotional exhaustion and psychological distress (3, 4). Despite a growing body of literature examining workplace conflicts in general nursing, the specific effects of these conflicts on MHNs remain under-researched (5). This study aims to address this gap by investigating the unique impact of interpersonal conflict on MHNs' emotional exhaustion and psychological distress.

As the largest component of the mental health workforce globally, MHNs are required to manage not only the psychiatric symptoms of their patients but also the intense emotional demands that accompany mental health care (1). These demands place MHNs at a disproportionately high risk for psychological distress compared to nurses in other specialties. The prevalence of psychological distress among nurses ranges from 17% to 49%, with MHNs likely experiencing the upper bounds of this range due to the emotionally charged environments in which they operate (6). For MHNs, this psychological burden is exacerbated by the necessity to constantly mediate challenging interpersonal dynamics and emotionally complex care scenarios (7). For MHNs, this psychological burden is exacerbated by the necessity to constantly mediate challenging interpersonal dynamics and emotionally complex care scenarios.

Emotional exhaustion, a core component of burnout, is defined as a state of emotional depletion resulting from prolonged exposure to high levels of occupational stress (8). Previous research indicates that 11.23% of nurses globally experience job burnout, primarily driven by severe emotional exhaustion (9, 10). Moreover, the prevalence of emotional exhaustion among psychiatric nurses can reach up to 28.1% (11). This elevated risk is primarily attributable to the intense emotional labor required in mental health care, where MHNs frequently encounter aggressive or emotionally unstable patients. The consequences of emotional exhaustion among MHNs are severe, encompassing both diminished personal wellbeing and compromised patient care outcomes, with far-reaching implications for healthcare delivery and workplace safety (10). Factors such as role ambiguity, excessive workload, workplace aggression, and conflicting job demands further exacerbate this risk (11). However, institutional support systems, including clinical supervision and robust social support networks, have been shown to mitigate the effects of emotional exhaustion, underscoring the importance of systemic interventions in preserving MHNs' wellbeing (12).

Interpersonal conflict is a pervasive issue within psychiatric nursing environments, where emotionally charged interactions are common. Conflicts centered around relationships, such as patient mistreatment, conflicts with supervisors and colleagues, or work-family conflicts, can have a range of detrimental effects if not managed effectively. Such conflicts, if inadequately addressed, have been linked to increased emotional exhaustion, heightened psychological distress, diminished job satisfaction, elevated turnover rates, and poorer patient outcomes (5). Despite the recognized importance of addressing these conflicts, there is a limited understanding of the specific types of interpersonal conflicts that most significantly contribute to emotional exhaustion and psychological distress in MHNs. Therefore, this study aims to elucidate these relationships by constructing a structural network that explores the intricate linkages between workplace interpersonal conflicts, emotional exhaustion, and psychological distress in the MHN workforce.

## 2 Methods

### 2.1 Design

A cross-sectional descriptive correlational survey design was utilized to assess interpersonal conflict and psychological distress among nurses in the ward. This study adhered to the guidelines of Strengthening the Reporting of Observational Studies in Epidemiology (STROBE) for observational research and reporting standards for psychological network analyses in cross-sectional data. Ethics approval for this research was obtained from the West China Hospital of Sichuan University (reference: 2023–1846).

### 2.2 Participants, sampling, and setting

We employed purposive sampling, a commonly used method for selecting nurse samples (13). The study's inclusion criteria required (a) participants to be registered nurses at the 15 centers contacted in Sichuan Province, China, (b) with a minimum of 3 months' tenure at the institution, and (c) their agreement to cooperate after detailed explanations from the research team. Exclusions encompassed (a) administrative staff, (b) nurses in non-inpatient settings (e.g., operating rooms, emergency departments, clinics), as well as (c) those on unpaid leave or study-related absence. Based on the assumption that 55.5% of participants have severe psychological distress according to previous research (14), with a margin of error of 5.5%, a 95% confidence interval, and a design effect of three due to non-probability sampling, the estimated minimum sample size is 924.

In this study, all nurses were recruited from six tertiary comprehensive hospitals or nine secondary psychiatric specialty hospitals across seven prefecture-level cities in Sichuan Province (comprising 18 prefecture-level cities), located in the southwestern region of China. These cities include the capital of Sichuan Province, Chengdu, as well as Nanchong City, Panzhihua City, Leshan City, Mianzhu City, Suining City, and Yibin City, areas where healthcare resources are relatively limited.

### 2.3 Data collection

Between March 30th and April 5th, 2024, online questionnaires were collected. Initially, 15 nursing department directors from six tertiary comprehensive hospitals and nine secondary psychiatric specialty hospitals were given access to the questionnaire. They were instructed to distribute it to head nurses in various clinical departments via WeChat groups, who then forwarded it to their respective departmental work groups. All participants volunteered and electronically signed informed consent forms before anonymously completing the questionnaires (submission required completion of all questions). Questionnaire quality was assessed by two trained postgraduates to ensure validity.

### 2.4 Measures

The online questionnaires consisted of three sections: demographic characteristics, workplace interpersonal conflicts, emotional exhaustion, and psychological distress.

#### 2.4.1 Demographic characteristics

Demographic characteristics in the study encompassed age (in years), gender (male, female), years of experience as a nurse (in years), department type (MHNs, medical/surgical nurses), professional title (primary, intermediate, senior), educational level (associate's degree or lower, bachelor's degree or higher), marital status (single, married/cohabitating, divorced/widowed), and subjective social status. Subjective social status was assessed using the MacArthur Scale of Subjective Social Status, which is the most widely used subjective social status scale (15). Participants were presented with a depiction of a ladder with 10 rungs, accompanied by the following description: “Think of this ladder as representing where people stand in our society. At the top of the ladder are the people who are the best off, those who have the most money, most education, and best jobs. At the bottom are the people who are the worst off, those who have the least money, least education, and worst jobs or no job.” Additionally, the measure demonstrated moderate test-retest reliability (Kappa = 0.62), and the structural validity of the MacArthur Scale of Subjective Social Status was robust (16).

#### 2.4.2 Interpersonal conflicts arising from patients, colleagues, and family

Patient mistreatment: patient mistreatment refers to unfair, hostile, and demeaning behavior from patients toward healthcare staff during the care process, excluding physical violence (17). The Customer Interpersonal Injustice scale was used to measure instances of patient mistreatment encountered by nurses in the workplace over the past 6–12 months (18). The scale comprises eight items, where participants assess their experiences using a 5-point Likert scale ranging from 1 (never) to 5 (frequently). Scores range from 5 to 40, where higher scores indicate greater perceived unfairness. The scale demonstrated robust internal consistency, with a Cronbach's α coefficient of 0.88 in previous studies (18). In this study, the Cronbach's α coefficient was calculated to be 0.939.

Interpersonal conflict arising from colleagues: the Interpersonal Conflict at Work Scale (ICAWS) was used to measure conflict experienced by nurses in the workplace with both supervisors (items 1–4; Conflict with coworker at work, CCW) and coworkers (items 5–8; Conflict with supervisor at work, CSW) over the past 6–12 months, totaling 8 items. Responses were rated on a 5-point Likert scale, with options ranging from 1 (Less than once per month or never) to 5 (Several times per day), and scoring was based on the average of the items. The scale's items were adapted from Spector and Jex's 4-item ICAWS (19), following suggestions by Frone to assess conflicts with supervisors and coworkers separately (20). The scale's reliability was validated by Liu et al. in China, with an overall Cronbach's α value of 0.87 (21).

Work-family conflict: the Bi-directional Scale of Work-Family Conflict (22), which includes the Work Interfering with Family (WIF) and Family Interfering with Work (FIW) subscales, was employed to assess the impact of work on family and family on work over the past 6–12 months. The WIF consists of six items, while the FIW comprises five items, both rated on a 7-point Likert scale ranging from 1 (Strongly disagree) to 7 (Strongly agree), where higher scores indicate higher levels of WIF or FIW. The Chinese version of the Work-Family Conflict Scale has been demonstrated to possess good reliability and validity (23). In this study, the overall Cronbach's α for the total scale was 0.924, with Cronbach's α coefficients of 0.957 for WIF and 0.772 for FIW.

#### 2.4.3 Emotional exhaustion

The emotional exhaustion sub-scale of the Maslach Burnout Inventory (MBI), consisting of six items, was used to measure nurses' emotional exhaustion at work over the past 6–12 months (24). The scale was rated on a 5-point Likert scale ranging from 1 (Strongly disagree) to 5 (Strongly agree), where a higher total score indicates a greater perception of emotional exhaustion. The Chinese version of the MBI has been validated for reliability and effectiveness among Chinese nurses (25). In this study, the Cronbach's α coefficient was calculated to be 0.942.

#### 2.4.4 Psychological distress

The 6-item Kessler Psychological Distress Scale (K6) was utilized to measure nurses' psychological distress over the most severe 30 days within the past 12 months. It employs a 5-point Likert scale ranging from 0 (none of the time) to 4 (All of the time). Total scores range from 0 to 24, with higher scores indicating more severe psychological distress. Scores of K6 ≥13 are considered indicative of severe psychological distress (26). The Chinese version of K6 has been previously validated in the WHO World Mental Health survey (27). In our study, the Cronbach's α coefficient was calculated to be 0.936.

### 2.5 Data analysis

All statistical analyses were performed with R Studio version 2023.06.0+421 and R version 4.4.0 between April and May 2024. We used the skimr package to check for missing data, and there is no missing data. Two-tailed *p*-values < 0.05 were considered statistically significant.

Descriptive statistics were employed to summarize the characteristics of the respondents. Percentages were reported for categorical variables, while means and standard deviations (SD) were reported for continuous variables. We assessed differences between MHNs and non-MHNs using Chi-square tests or Fisher's exact test for categorical demographic variables and independent sample *t*-tests for continuous variables. We compared differences in the workplace interpersonal conflicts, emotional exhaustion, and psychological distress between MHNs and non-MHNs using independent sample *t*-tests. Bivariate associations were analyzed between demographic characteristics and both emotional exhaustion and psychological distress, as well as between workplace interpersonal conflicts and these two outcomes, using independent sample *t*-tests or one-way ANOVA for group comparisons, and Pearson correlation for continuous variables.

#### 2.5.1 Multiple regression analysis

Before network construction, multiple regression analyses were conducted to identify factors associated with emotional exhaustion and psychological distress, incorporating demographic characteristics, and interpersonal conflicts as predictor variables. Forward stepwise selection determined the final explanatory variables for the linear regression models. Additionally, differences in emotional exhaustion and psychological distress attributed to demographic characteristics and workplace interpersonal conflicts were examined through a multiple linear regression model, ensuring the absence of multicollinearity with tolerance ranging from 0.39 to 0.913 and variance inflation factor (VIF) from 1.047 to 2.567. For severe psychological distress, defined by a K6 score ≥13, forward stepwise selection based on the likelihood ratio criterion was used to identify explanatory variables for the logistic regression model.

#### 2.5.2 Network analysis

We used the qgraph package to construct networks (28) representing workplace conflicts, emotional exhaustion, and psychological distress among nurses. The NetworkComparisonTest package was employed to compare network differences between MHNs and non-MHNs. Network estimation was performed using the bootnet package, where we applied the Least Absolute Shrinkage and Selection Operator (LASSO) to shrink coefficients, combined with the Extended Bayesian Information Criterion (EBIC) to evaluate model quality (29). A tuning parameter gamma was set to 0.5 to find the optimal penalty parameter for LASSO, thereby obtaining the best model (30).

For network visualization, we used the Fruchterman-Reingold algorithm with the “spring” layout (31). In the visualized networks, nodes with stronger connections were positioned closer to the center, while those with weaker connections were located on the periphery. In the network, each node represented a variable, and edges indicated correlations between variables, with green edges representing positive correlations and red edges representing negative correlations (32). The thickness of the edges reflects the strength of the connections between nodes (33).

Centrality indices (i.e., strength, closeness, and betweenness) were evaluated using the qgraph package (32). Strength centrality was defined as the sum of the absolute weights of edges connected to each node, representing the node's ability to influence other symptoms. Higher strength centrality indicates a greater likelihood of co-occurrence with other symptoms. Closeness centrality was defined as the inverse of the shortest path lengths from one symptom node to all other nodes, indicating the node's central position in the network. Higher closeness centrality implies a shorter overall path length to other nodes. Betweenness centrality measured the shortest path length connecting any two nodes, reflecting the node's role in bridging connections within the network. Nodes with higher betweenness centrality have a greater impact on the network. Among these indices, strength centrality was considered the most important and reliable (30).

To assess network accuracy and stability, we used the bootnet package to perform bootstrapping with 19,000 bootstrap samples (30). A bootstrapped difference test was conducted to evaluate centrality accuracy. Edge differences were tested using an edge difference plot, where edges with 95% bootstrapped confidence intervals crossing the zero line were shaded gray, while those with significant differences (i.e., not crossing zero) were shaded black (34). The correlation stability coefficient was used to assess the stability of centrality measures. It was estimated as the maximum proportion of cases that can be dropped while still maintaining a correlation of 0.7 in the ranking of centrality measures (30). Stability coefficients were expected to be at least >0.25, with a preferred threshold of >0.5 (35).

## 3 Results

### 3.1 Characteristics and prevalence of psychological distress

**Figure 1:**
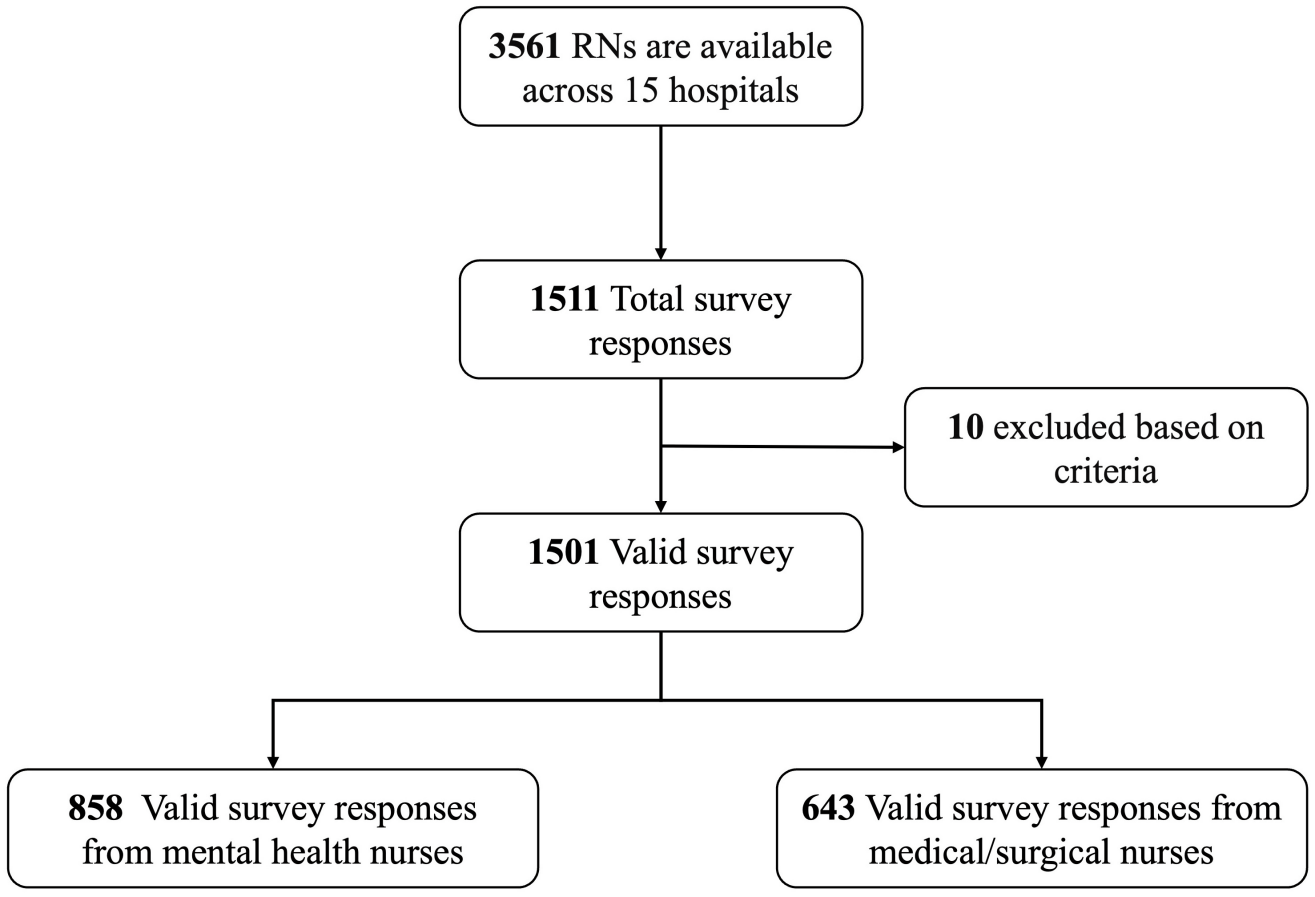
Flowchart of sampling procedure.

In total, we consecutively received responses from 1,511 out of 3,561 registered nurses (42.2%), with 1,501 valid surveys completed (99.3%). Of these, 858 responses (57.2%) were from MHNs and 643 (42.8%) were from non-MHNs (see flowchart in Figure 1). The participants had a mean age of 32.22 years (SD = 12.75) and an average nursing experience of around 10.08 years (SD = 7.20). Significant demographic differences, along with differences in perceived patient mistreatment, conflicts with supervisors and colleagues, and work-family conflicts, were noted between MHNs and non-MHNs, as shown in the participant profile in Table 1. Overall, 5.1% of participants had severe psychological distress.

**Table 1:** Characteristics of study participants.

	**Total (*N* = 1,501)**	**Mental health nurses (*n* = 858)**	**Medical/surgical nurses (*n* = 643)**	***P-value***
Gender, *n* (%)				<0.001^*^
Female	1,352 (90.1)	726 (84.6)	626 (97.4)	
Male	149 (9.9)	132 (15.4)	17 (2.6)	
Professional title, *n* (%)				<0.001^*^
Primary	1,002 (66.8)	624 (72.7)	378 (58.8)	
Intermediate	437 (29.1)	217 (25.3)	220 (34.2)	
Senior	62 (4.1)	17 (2.0)	45 (7.0)	
Educational level, *n* (%)				<0.001^*^
Associate's degree or under	484 (32.2)	327 (38.1)	157 (24.4)	
Bachelor's degree or above	1,017 (67.5)	531 (61.9)	486 (75.6)	
Marital status, *n* (%)				0.014^*^
Single	455 (30.3)	283 (33.0)	172 (26.7)	
Married/cohabitating	994 (66.2)	551 (64.2)	443 (68.9)	
Divorced/widowed	52 (3.5)	24 (2.8)	28 (4.4)	
Subjective social status^†^	4.78 (1.85)	4.55 (1.86)	5.08 (1.80)	<0.001^*^
Interpersonal conflicts				
Patient mistreatment^†^	12.35 (5.11)	12.19 (5.00)	12.55 (5.24)	0.178
ICAWS overall scores^†^	1.12 (0.32)	1.12 (0.33)	1.12 (0.31)	0.975
Conflict with supervisor^†^	1.12 (0.36)	1.12 (0.36)	1.13 (0.36)	0.574
Conflict with coworker^†^	1.12 (0.35)	1.13 (0.35)	1.12 (0.33)	0.521
WIF scores^†^	21.75 (9.73)	21.71 (9.29)	21.79 (10.29)	0.876
FIW scores^†^	12.39 (5.39)	12.74 (5.50)	11.91 (5.21)	0.003^*^
Emotional exhaustion				
Emotional exhaustion scale scores^†^	15.02 (5.88)	15.33 (5.70)	14.62 (6.09)	0.021^*^
Psychological distress				
K6 scores^†^	4.65 (4.48)	4.87 (4.64)	4.35 (4.25)	0.026^*^
Severe psychological distress (K6 ≥ 13), *n* (%)	77 (5.1)	55 (6.4)	22 (3.4)	0.009^*^

^*^Significant at *P* < 0.05. † Mean and standard deviations.

ICAWS, the Interpersonal Conflict at Work Scale; WIF, Work Interfering with Family from the Bi-directional Scale of Work-Family Conflict; FIW, Family Interfering with Work from the Bi-directional Scale of Work-Family Conflict; K6, the 6-item Kessler Psychological Distress Scale.

There is no difference between demographic characteristics and emotional exhaustion or psychological distress, except that male nurses score higher than female nurses on K6 scores (Table 2). The subjective social status scores are negatively correlated with emotional exhaustion (*r* = −0.25, *p* < 0.001) and K6 scores (*r* = −0.21, *p* < 0.001). Emotional exhaustion and K6 scores are positively correlated with scores of interpersonal conflicts at work (*p* < 0.001).

**Table 2:** Bivariate associations between participant characteristics and measures of emotional exhaustion and psychological distress.

**Factors**	**Emotional exhaustion scale scores [Mean (SD)]**	***P-value***	**Kessler psychological distress scale scores [Mean (SD)]**	***P-value***
Age^†^	−0.019	0.472	−0.037	0.153
Gender		0.222		0.002
Female	14.91 (5.85)		4.54 (4.35)	
Male	15.99 (6.03)		5.61 (5.45)	
Years as nurse^†^	−0.016	0.543	−0.034	0.184
Professional title		0.081		0.722
Primary	15.02 (5.86)		4.68 (4.57)	
Intermediate	15.23 (5.93)		4.75 (4.41)	
Senior	13.65 (5.71)		3.50 (3.23)	
Educational level		0.996		0.283
Associate's degree or under	14.88 (5.78)		4.59 (4.57)	
Bachelor's degree or above	15.09 (5.93)		4.68 (4.44)	
Marital status		0.091		0.622
Single	15.13 (6.05)		4.60 (4.60)	
Married/cohabitating	15.01 (5.78)		4.70 (4.44)	
Divorced/widowed	14.33 (6.25)		4.04 (4.18)	
Subjective social status^†^	−0.248	<0.001	−0.209	<0.001
Patient mistreatment^†^	0.446	<0.001	0.476	<0.001
Conflict with supervisor^†^	0.289	<0.001	0.359	<0.001
Conflict with coworker^†^	0.278	<0.001	0.352	<0.001
Work-to-family conflict^†^	0.693	<0.001	0.439	<0.001
Family-to-work conflict^†^	0.402	<0.001	0.497	<0.001

^†^Results were presented using the Pearson correlation coefficient.

### 3.2 Risk and protective factors association with emotional exhaustion and psychological distress

**Figure 2:**
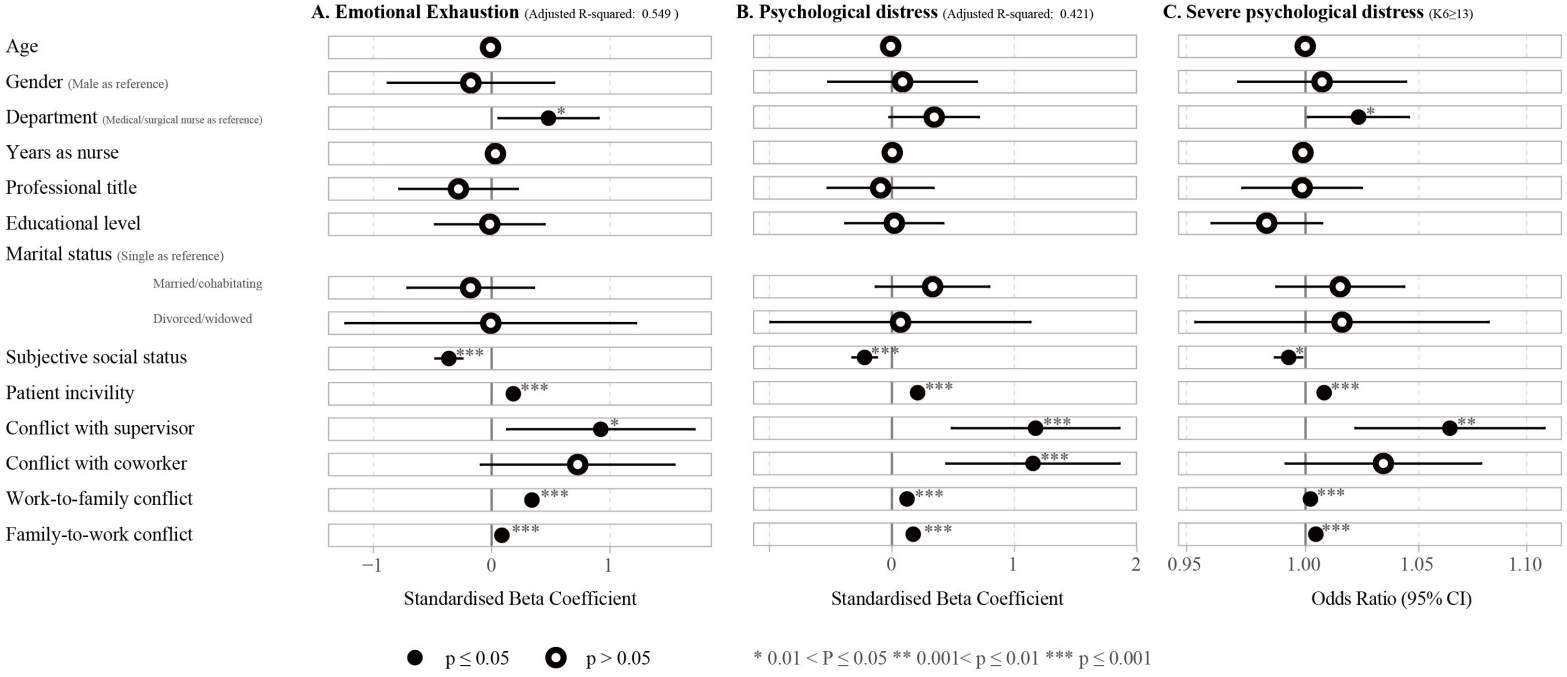
Forest plots of multivariate regression analyses showing associations between participant characteristics and **(A)** emotional exhaustion (Adjusted R-squared: 0.549) and **(B)** psychological distress based on linear regression models (Adjusted R-squared: 0.421), and **(C)** severe psychological distress based on logistic regression models.

Multinomial regression analysis showed that higher subjective social status is associated with lower emotional exhaustion and psychological distress, including severe psychological distress (Figure 2; see more detail in Supplementary Table 1). On the contrary, being a MHN, along with higher scores in patient mistreatment, CSW, and WIF and FIW conflicts, are key contributors to elevated emotional exhaustion. Additionally, higher scores in patient mistreatment, conflicts with supervisors and coworkers, as well as WIF and FIW conflicts, are associated with increased K6 scores. These factors, particularly being a MHN and experiencing higher levels of patient mistreatment and work-family conflicts, are high-risk indicators for severe psychological distress.

### 3.3 Overall network

#### 3.3.1 Density and centrality of networks

**Figure 3:**
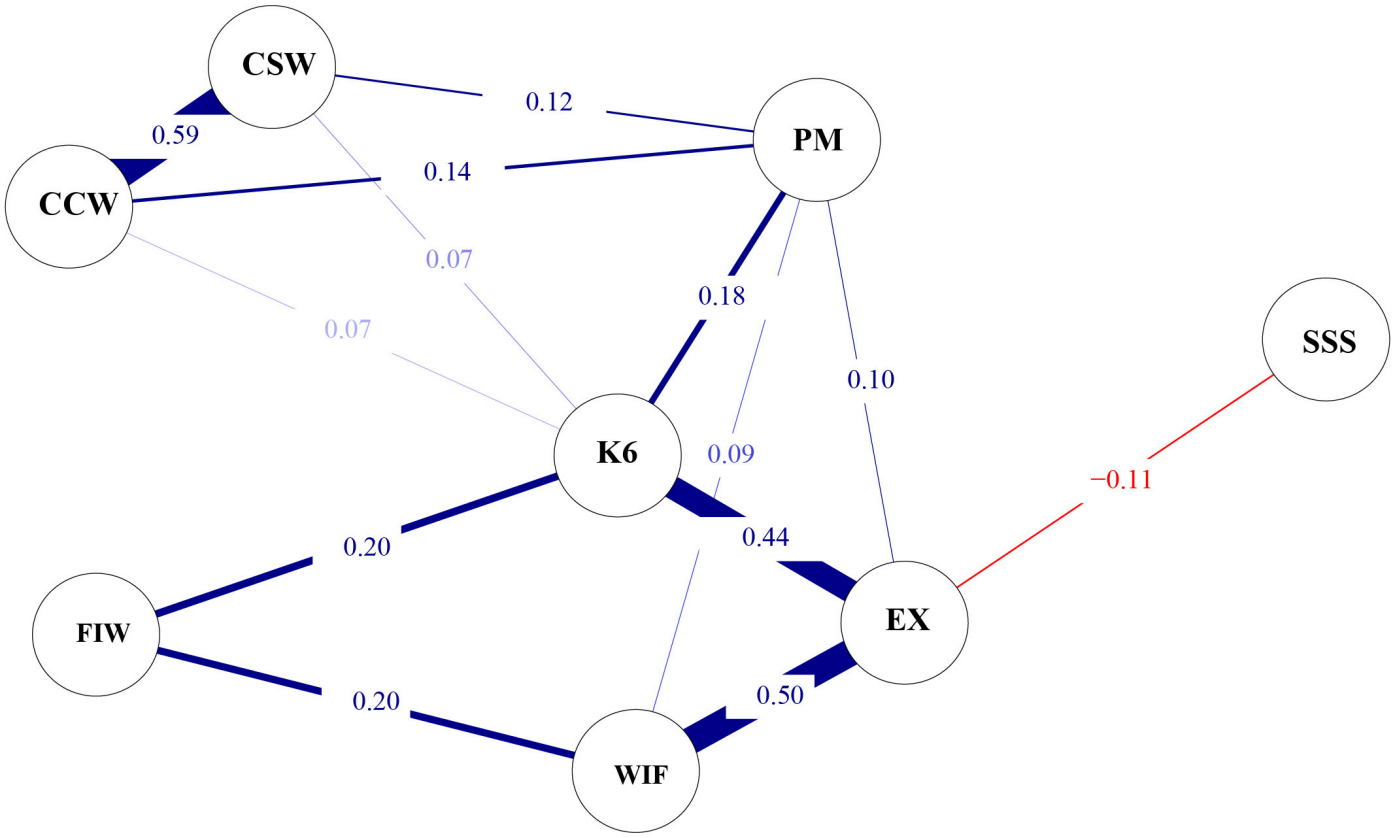
Regularized partial correlation network (*n* = 1,501). K6, 6-item Kessler Psychological Distress Scale; EX, Emotional Exhaustion Scale; SSS, MacArthur Scale of Subjective Social Status; PM, Patient Mistreatment; CSW, Conflict with Supervisor at Work; CCW, Conflict with Coworker at Work; WIF, Work Interfering with Family from the Bidirectional Scale of Work-Family Conflict; FIW, Family Interfering with Work from the Bidirectional Scale of Work-Family Conflict.

The network structure among subjective social status, interpersonal conflicts at work, emotional exhaustion, and psychological distress is illustrated in Figure 3. The network density was 0.679 (19/28 edges), with a mean weight of 0.093. There were strong connections between work-to-family conflict and emotional exhaustion (*r* = 0.50), as well as between emotional exhaustion and psychological distress (*r* = 0.44). Additionally, psychological distress was weakly associated with conflicts with supervisors (*r* = 0.07) and coworkers (*r* = 0.07).

#### 3.3.2 Network comparison between MHNs and non-MHNs

**Figure 4:**
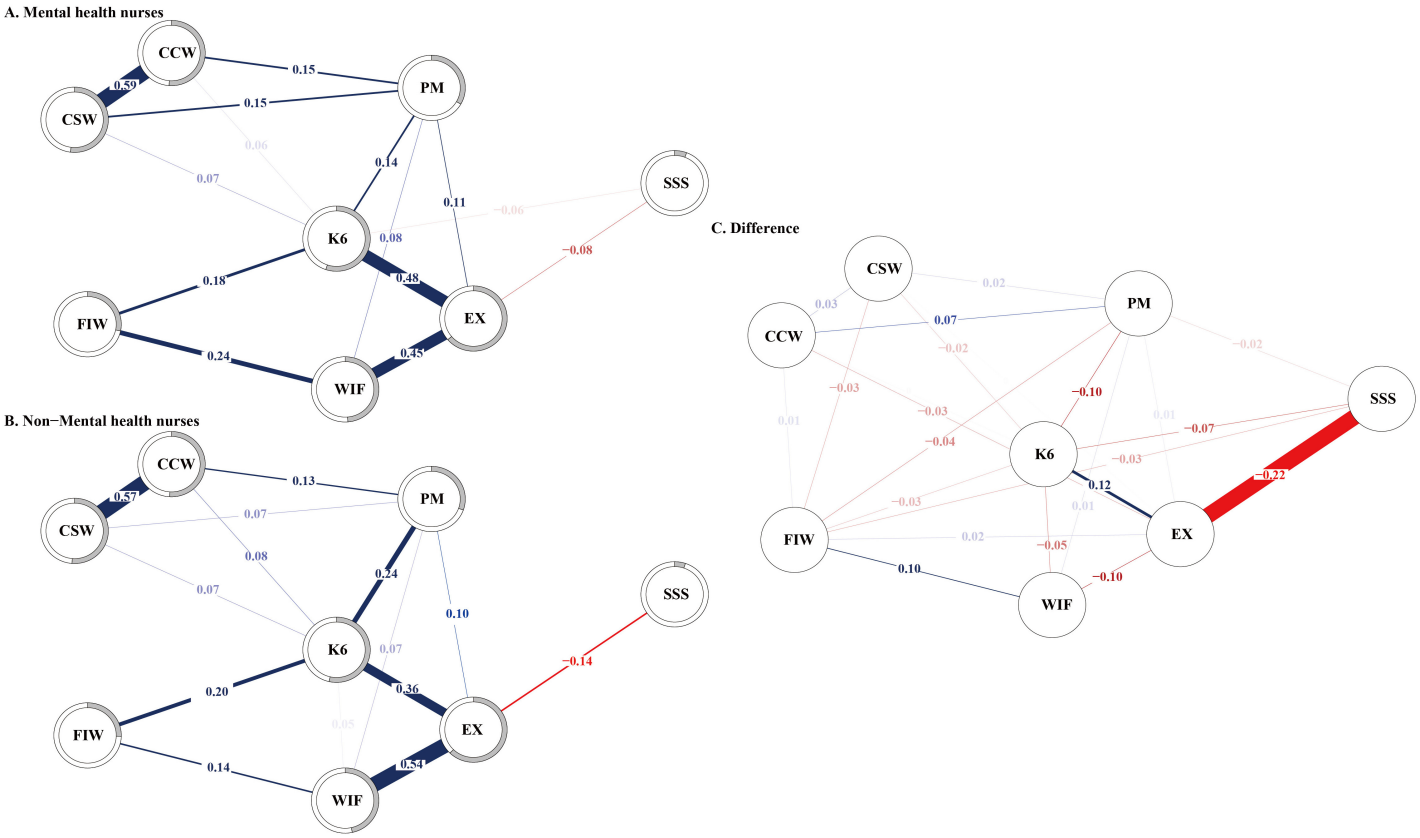
Regularized partial-correlation networks for **(A)** the mental health nurses (*n* = 858), **(B)** the non-mental health nurses (*n* = 643), and **(C)** difference between the two. K6, 6-item Kessler Psychological Distress Scale; EX, Emotional Exhaustion Scale; SSS, MacArthur Scale of Subjective Social Status; PM, Patient Mistreatment; CSW, Conflict with Supervisor at Work; CCW, Conflict with Coworker at Work; WIF, Work Interfering with Family from the Bidirectional Scale of Work-Family Conflict; FIW, Family Interfering with Work from the Bidirectional Scale of Work-Family Conflict.

Figure 4 illustrates the network for MHNs and non-MHNs, along with their differences. Network comparison tests revealed that the overall network structure did not differ significantly between the MHNs and non-MHNs' networks (*p* = 0.937). However, significant differences were observed in the connections between emotional exhaustion and psychological distress (*p* = 0.003, difference = 0.12), emotional exhaustion and WIF conflict (*p* = 0.029, difference = −0.10), psychological distress and WIF conflict (*p* = 0.046, difference = −0.05), as well as patient mistreatment and FIW conflict (*p* = 0.025, difference = −0.04), despite these differences being relatively minor.

#### 3.3.3 Node centrality

**Figure 5:**
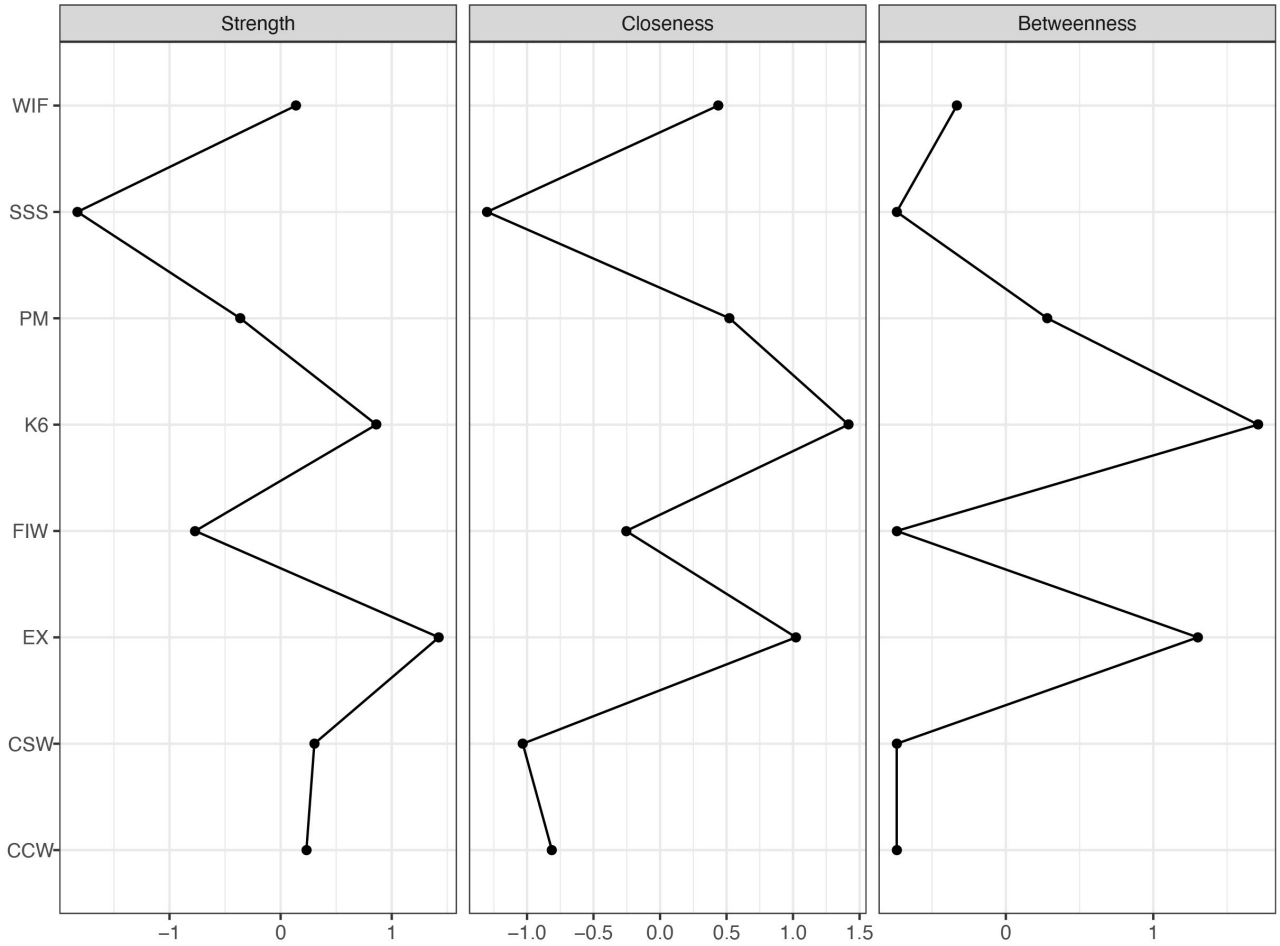
Centrality indices of strength, closeness, and betweenness (*n* = 1,501). K6, 6-item Kessler Psychological Distress Scale; EX, Emotional Exhaustion Scale; SSS, MacArthur Scale of Subjective Social Status; PM, Patient Mistreatment; CSW, Conflict with Supervisor at Work; CCW, Conflict with Coworker at Work; WIF, Work Interfering with Family from the Bidirectional Scale of Work-Family Conflict; FIW, Family Interfering with Work from the Bidirectional Scale of Work-Family Conflict.

Figure 5 illustrates the strength, betweenness, and closeness centrality metrics for the overall network. Our centrality analysis revealed that psychological distress (*r*_*s*_ = 1.42), emotional distress (*r*_*s*_ = 0.86), and CSW (*r*_*s*_ = 0.30) exhibited the highest strength values. Furthermore, psychological distress (*r*_*b*_ = 1.30, *r*_*c*_ = 1.42), emotional distress (*r*_*b*_ = 1.71, *r*_*c*_ = 1.02), and patient mistreatment (*r*_*b*_ = 0.28, *r*_*c*_ = 0.52) showed the largest betweenness and closeness centrality values.

#### 3.3.4 Accuracy and stability of networks

**Figure 6:**
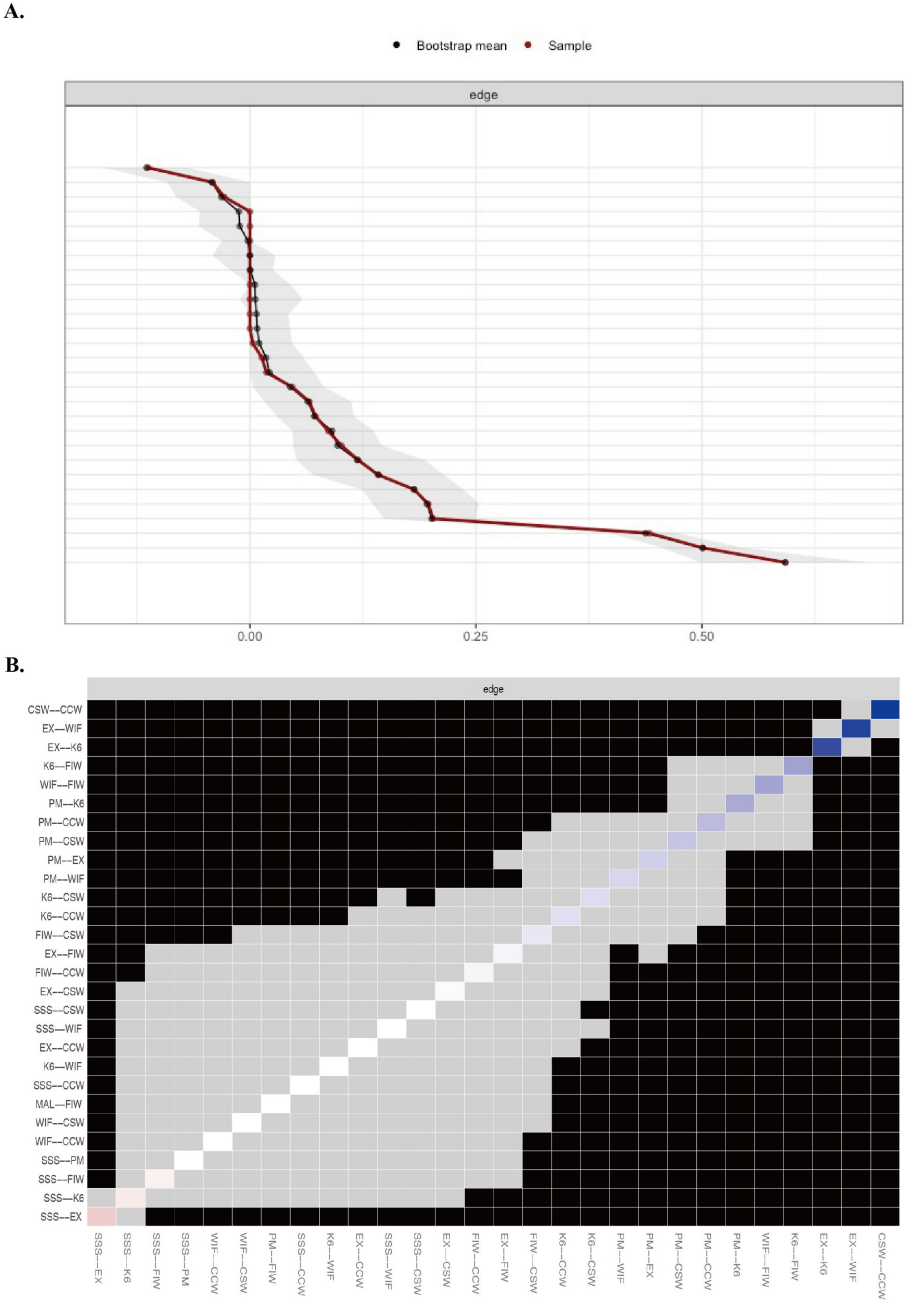
Bootstrap analysis results of the **(A)** edge weight and **(B)** difference between edges. K6, 6-item Kessler Psychological Distress Scale; EX, Emotional Exhaustion Scale; SSS, MacArthur Scale of Subjective Social Status; PM, Patient Mistreatment; CSW, Conflict with Supervisor at Work; CCW, Conflict with Coworker at Work; WIF, Work Interfering with Family from the Bidirectional Scale of Work-Family Conflict; FIW, Family Interfering with Work from the Bidirectional Scale of Work-Family Conflict.

In general, the confidence intervals were narrow, indicating stable results (Figure 6A). As shown in Figure 6B, the black squares between the following pairs: CSW-CCW and CSW-patient mistreatment, CCW-CSW and CCW-patient mistreatment, CSW-CCW and CSW-K6, CCW-CSW and CCW-K6, emotional exhaustion-K6 and emotional exhaustion-subjective social status, K6-emotional exhaustion and K6-FIW, K6-emotional exhaustion and K6-patient mistreatment, K6-emotional exhaustion and K6-CSW, K6-emotional exhaustion and K6-CCW, emotional exhaustion-subjective social status and emotional exhaustion-WIF, WIF-emotional exhaustion and WIF-FIW, and patient mistreatment-emotional exhaustion and patient mistreatment-WIF, indicate statistically significant differences between these nodes. The correlation stability coefficient for strength centrality and closeness is 0.85, indicating strong stability in their results. Additionally, the correlation stability coefficient for betweenness is 0.45, indicating that betweenness is stable.

## 4 Discussion

To our knowledge, this is the first network analysis study that has constructed a network structure linking interpersonal conflict in the workplace with emotional exhaustion and psychological distress among nurses, and explored the points of contact where interpersonal conflict affects the network of emotional exhaustion and psychological distress. In this study, 5.1% of the nurses overall experienced severe psychological distress. The proportion of severe psychological distress was significantly higher among MHNs compared to non-MHNs. Emotional exhaustion among nurses was not only related to being an MHN, but also associated with lower subjective social status, more severe conflict with patients and supervisors, and bidirectional work-family conflict. More severe psychological distress among nurses was linked to lower subjective social status, more severe conflict with patients and supervisors, and bidirectional work-family conflict. Risk factors for severe psychological distress included being an MHN, patient mistreatment, and bidirectional work-family conflict, while a protective factor was higher subjective social status. The network analysis revealed no significant differences in the network structures of interpersonal conflict, emotional exhaustion, and psychological distress between MHNs and non-MHNs. Within the emotional exhaustion node, work-to-family conflict had the greatest impact, followed by psychological distress, with subjective social status showing a negative correlation. In the psychological distress node, emotional exhaustion had the greatest impact, followed by family-to-work conflict, patient mistreatment, and conflict with colleagues and supervisors.

In line with previous research (36), the low prevalence of severe psychological distress among nurses in this study may be due to the K6 scale's specific design to distinguish between severe mental illness and non-cases. Additionally, consistent with earlier findings (1, 37), this study confirmed a close association between nurses' psychological distress and emotional exhaustion. In addition, this study found that MHNs exhibited higher levels of emotional exhaustion and psychological distress in the workplace compared to non-MHNs. Furthermore, MHNs were at a higher risk of experiencing severe psychological distress than those working in medical or surgical departments. This suggests that government agencies, the public, and hospital administrators should pay greater attention to the heightened emotional exhaustion and psychological distress experienced by MHNs in comparison to their counterparts in surgical or medical nursing. Consistent with previous research, this study found a significant negative association between subjective social status and mental health outcomes (38). Nurses' perceptions of their social standing may be shaped by multiple factors, including income level, societal recognition, and the perceived prestige of the nursing profession (39). Among the participants in our study, nurses reported generally low levels of subjective social status, with MHNs perceiving significantly lower status compared to non-MHNs. Given the heightened vulnerability of MHNs, targeted efforts are urgently needed to enhance their societal recognition and professional standing. In addition to strengthening media representation and increasing MHNs' engagement in healthcare policymaking, it is crucial for nurses themselves, across all specialties, to proactively shape and promote a positive professional image (40). Strategic initiatives addressing both societal attitudes and institutional structures are essential not only for improving the overall subjective social status of nurses, but also for meeting the specific needs of MHNs and supporting their mental health.

Emotional labor entails the regulation of personal emotions to provide effective care for others while safeguarding one's own emotional wellbeing (41, 42). Given the inherently interpersonal nature of nursing work, nurses frequently encounter conflicts that require the management of their emotional responses to uphold professional standards (43). In the context of interpersonal conflict in the workplace, our findings are consistent with previous research, indicating that work-family conflict is not only closely associated with emotional exhaustion (3, 44, 45) but also significantly linked to psychological distress (4, 46). Work-family conflict typically results in emotional exhaustion and psychological distress, which in turn can further intensify the conflict (47, 48). Working hours appear to be a significant determinant of work-family conflict (49). Nurses who work <50% of the time or have flexible schedules are afforded more time for family and personal life, which can help reduce emotional exhaustion (44). To address work-family conflict and its adverse outcomes, studies have suggested that alternative work arrangements, such as flexible working hours and workload sharing, may alleviate conflict, while supportive supervisory behaviors and organizational support can mitigate its negative consequences (50).

Additionally, our study revealed that nurses' experiences of patient mistreatment and conflicts with supervisors are both linked to their emotional exhaustion, while patient mistreatment, conflicts with supervisors, and conflicts with coworkers are all significantly associated with their psychological distress. The positive association between customer mistreatment and emotional exhaustion highlights that (51), for nurses, patients are effectively their clients, and the nurse-patient relationship is one of the most common interpersonal dynamics in hospitals. Patient mistreatment is a widespread issue (52), and emotional exhaustion among nurses is linked to declines in care quality, patient safety, patient satisfaction, and nurse productivity (53). Interpersonal conflicts in the workplace, including conflicts with supervisors and coworkers, are consistently associated with psychological distress. This distress could arise from the inherent power imbalances in these relationships or from the emotional toll of managing such conflicts in already high-stress environments (5). Addressing these issues through management-level interventions is critical. These interventions should focus on reducing patient mistreatment, fostering healthy supervisor-employee relationships, and improving overall team dynamics. Enhancing supervisory support can play a key role in alleviating emotional exhaustion and psychological distress, creating a healthier work environment that promotes both nurse wellbeing and patient care outcomes (54).

Our network analysis results revealed no significant differences in the structural network of interpersonal conflicts associated with emotional exhaustion and psychological distress between MHNs and non-MHNs. This suggests that, despite the distinct work environments and patient populations they serve, both groups experience similar patterns of interpersonal conflict. Notably, work-to-family conflict emerged as a central interpersonal issue for nurses across both groups, strongly linked to their emotional exhaustion and psychological distress. This highlights the importance of addressing work-life balance in nursing, as unresolved work-to-family conflicts can exacerbate stress and burnout, ultimately impacting both nurses' wellbeing and the quality of care they provide (50). Addressing these challenges requires systemic changes informed by nurses' lived experiences. It is essential to more closely link nurses' voices with tangible changes in the work environment. Actively listening to nurses' perspectives on work-life balance could help guide the development of evidence-based interventions aimed at improving working conditions (55). Such efforts would support a healthier integration of nurses' professional and personal lives and help them realize their full potential while safeguarding their mental health.

This study employed a cross-sectional design to survey nurses in Sichuan Province, China, which limits the ability to draw causal inferences between variables. Additionally, the generalizability of the findings may be constrained due to the specific population studied. Moreover, the comparison in this study was only between MHNs and non-MHNs, without considering other departments in general hospitals or further subdividing nursing subspecialties. The research focused solely on the relationship between workplace interpersonal conflicts and nurses' emotional exhaustion and psychological distress, without capturing the influence of other workplace-related stressors or factors. Future research should consider incorporating other workplace stressors and employing prospective longitudinal designs to better assess causality.

## 5 Conclusions

This study reveals that workplace interpersonal conflict significantly impacts emotional exhaustion and psychological distress among nurses. Elevated levels of both emotional exhaustion and psychological distress are associated with severe workplace conflicts, including bidirectional work-family conflict, patient mistreatment, and conflicts with supervisors and coworkers. Notably, MHNs experience greater psychological distress compared to their non-MHN counterparts. The analysis identifies work-to-family conflict as a major contributor to emotional exhaustion, which, in turn, strongly predicts psychological distress. These findings highlight the urgent need for targeted interventions to mitigate these stressors and enhance nurses' overall wellbeing.

## Data Availability

The raw data supporting the conclusions of this article will be made available by the authors, without undue reservation.
