# Supplementary material for: Network analysis of interpersonal conflict, emotional exhaustion and psychological distress among mental health nurses in the workplace: a cross-sectional survey
Source: Front Public Health. 2025 May 30;13:1559351. doi: 10.3389/fpubh.2025.1559351 (PMC12162648; doi:10.3389/fpubh.2025.1559351)
Supplement: Supplementary file 1 [file Table_1.docx]

Supplementary Material

# Supplementary Table

Supplementary Table 1 Multivariate Analysis of a)Linear regression models for the associations with participant characteristics and measures of emotional exhaustion (Model 1) and psychological distress (Model 2); b)logistic regression models for the for the associations with participant characteristics and severe mental illness (Model 3)

| **Parameter** | **Comparator vs Reference** | **Model 1: Emotional exhaustion** | | **Model 2: Psychological distress** | | **Model 3: Severe psychological distress** | |
| --- | --- | --- | --- | --- | --- | --- | --- |
|  |  | Coefficient (95%CI) | P-value | Coefficient (95%CI) | P-value | OR (95%CI) | P-value |
| Intercept |  | 4.77 (2.84 to 4.77) | <0.001 | -4.53 (-6.19 to -2.86) | <0.001 | -8.39 (-11.73 to -5.05) | <0.001 |
| Age |  | -0.10 (-0.03 to -0.01) | 0.251 | -0.01 (-0.03 to 0.01) | 0.222 | -0.03 (-0.13 to 0.08) | 0.595 |
| Gender | Female vs Male | -0.17 (-0.88 to -0.17) | 0.626 | 0.09 (-0.52 to 0.69) | 0.779 | 0.24 (-0.57 to 1.05) | 0.563 |
| Department | Mental health vs Non-mental health^†^ | 0.48 (0.06 to 0.48) | 0.026 | 0.34 (-0.02 to 0.71) | 0.065 | 0.84 (0.22 to 1.46) | 0.008 |
| Years as nurse |  | 0.03 (-0.01 to 0.03) | 0.163 | 0.00 (-0.03 to 0.04) | 0.854 | 0.00 (-0.11 to 0.10) | 0.952 |
| Professional title | others vs Primary | -0.28 (-0.78 to -0.28) | 0.272 | -0.09 (-0.52 to 0.34) | 0.674 | -0.21 (-0.97 to 0.54) | 0.572 |
| Educational level | Bachelor's degree or above vs Associate's degree or under | -0.02 (-0.48 to -0.02) | 0.947 | 0.02 (-0.38 to 0.42) | 0.926 | -0.50 (-1.11 to 0.01) | 0.105 |
| Marital status | Married/cohabitating vs Single | -0.18 (-0.71 to -0.18) | 0.514 | 0.33 (-0.13 to 0.79) | 0.160 | 0.43 (-0.31 to 1.17) | 0.251 |
|  | Divorced/widowed vs Single | -0.01 (-1.24 to -0.01) | 0.990 | 0.07 (-0.99 to 1.13) | 0.898 | 0.20 (-1.59 to 1.99) | 0.827 |
| MacArthur scale of subjective social status |  | -0.36 (-0.47 to -0.36) | <0.001 | -0.22 (-0.32 to -0.12) | <0.001 | -0.21 (-0.35 to -0.06) | 0.005 |
| Patient mistreatment |  | 0.18 (0.14 to 0.18) | <0.001 | 0.21 (0.17 to 0.25) | <0.001 | 0.12 (0.07 to 0.16) | <0.001 |
| Conflict with supervisor |  | 0.92 (0.13 to 0.92) | 0.022 | 1.17 (0.49 to 1.86) | <0.001 | 0.51 (-0.18 to 1.20) | 0.145 |
| Conflict with coworker |  | 0.73 (-0.09 to 0.73) | 0.081 | 1.15 (0.44 to 1.86) | 0.001 | 0.05 (-0.68 to 0.79) | 0.890 |
| Work-to-family conflict |  | 0.34 (0.32 to 0.34) | <0.001 | 0.12 (0.10 to 0.14) | <0.001 | 0.11 (0.07 to 0.16) | <0.001 |
| Family-to-work conflict |  | 0.09 (0.04 to 0.09) | <0.001 | 0.17 (0.14 to 0.21) | <0.001 | 0.10 (0.05 to 0.15) | <0.001 |

^†^ The non-mental health department encompasses disciplines such as medicine and surgery.
